# Susceptibility of microbial cells to the modified PIP_2_-binding sequence of gelsolin anchored on the surface of magnetic nanoparticles

**DOI:** 10.1186/s12951-019-0511-1

**Published:** 2019-07-08

**Authors:** Robert Bucki, Katarzyna Niemirowicz-Laskowska, Piotr Deptuła, Agnieszka Z. Wilczewska, Paweł Misiak, Bonita Durnaś, Krzysztof Fiedoruk, Ewelina Piktel, Joanna Mystkowska, Paul A. Janmey

**Affiliations:** 1 Department of Medical Microbiology and Nanobiomedical Engineering, Medical University of Bialystok, Mickiewicza 2c, 15-222 Białystok, Poland; 2 Institute of Chemistry, University of Białystok, Ciołkowskiego 1K, 15-245 Białystok, Poland; 3 Department of Microbiology and Immunology, The Faculty of Medicine and Health Sciences of the Jan Kochanowski University in Kielce, Aleja IX Wieków Kielc, 25-317 Kielce, Poland; 4 Department of Microbiology, Medical University of Bialystok, 15-222, Białystok, Poland; 5 Department of Materials Engineering and Production, Faculty of Mechanical Engineering, Bialystok University of Technology, Wiejska 45C, 15-351 Białystok, Poland; 6 Department of Physiology and Institute for Medicine and Engineering, University of Pennsylvania, Philadelphia, PA USA

**Keywords:** Gelsolin, PBP10 peptide, Magnetic nanoparticles, Fungal cells, Antibacterial

## Abstract

**Background:**

Magnetic nanoparticles (MNPs) are characterized by unique physicochemical and biological properties that allow their employment as highly biocompatible drug carriers. Gelsolin (GSN) is a multifunctional actin-binding protein involved in cytoskeleton remodeling and free circulating actin sequestering. It was reported that a gelsolin derived phosphoinositide binding domain GSN 160–169, (PBP10 peptide) coupled with rhodamine B, exerts strong bactericidal activity.

**Results:**

In this study, we synthesized a new antibacterial and antifungal nanosystem composed of MNPs and a PBP10 peptide attached to the surface. The physicochemical properties of these nanosystems were analyzed by spectroscopy, calorimetry, electron microscopy, and X-ray studies. Using luminescence based techniques and a standard killing assay against representative strains of Gram-positive (*Staphylococcus aureus* MRSA Xen 30) and Gram-negative (*Pseudomonas aeruginosa* Xen 5) bacteria and against fungal cells (*Candida* spp.) we demonstrated that magnetic nanoparticles significantly enhance the effect of PBP10 peptides through a membrane-based mode of action, involving attachment and interaction with cell wall components, disruption of microbial membrane and increased uptake of peptide. Our results also indicate that treatment of both planktonic and biofilm forms of pathogens by PBP10-based nanosystems is more effective than therapy with either of these agents alone.

**Conclusions:**

The results show that magnetic nanoparticles enhance the antimicrobial activity of the phosphoinositide-binding domain of gelsolin, modulate its mode of action and strengthen the idea of its employment for developing the new treatment methods of infections.

## Introduction

In the past decade, surface modified magnetic nanoparticles composed of an iron oxide core and a metallic or polymeric shell have received attention as potential nanomaterials that might be used in theranostic applications, for example as drug transporters, inducers of magnetic hyperthermia, MRI contrast agents and separators and trackers of macromolecules [1–4]. Many studies have focused on the development of an efficient and repeatable synthesis process to obtain size- and shape-controlled monodisperse and biocompatible nanoparticles [5]. For this purpose, different methods such as co-precipitation, thermal decomposition, microemulsion, sol–gel synthesis, and green synthesis using plant or bacterial extracts have been proposed [6]. Most of the previous work was directed to obtain a nanostructure with a specific shape via one-pot synthesis [7–9].

The physicochemical properties of nanomaterials exert a strong impact on their biomedical applications and therapeutic properties. Such parameters as size, shape and surface charge determine nanoparticle stability and dispersity which in turn regulate biological effects and influence surface interactions, cellular uptake and delivery processes [10]. Nanoparticle surface chemistry partially defines their interaction with bioactive agents used during the functionalization process as well as their biological effects [11, 12]. The conjugation process might be realized via physical or chemical interactions. Attachment-based physicochemical phenomena include ionic and hydrophobic interactions and dative bonding, which engages gold surface conduction electrons. Chemisorption via thiol derivatives can occur by the bifunctional linker carbodiimide (EDC)/active esters (NHS—*N*-hydroxy-succinimide) or by adapter molecules like avidin and biotin [13, 14]. In the case of a terminal amine group present on the surface, non-covalent (hydrogen bonding, ionic, electrostatic) and covalent (imine, enamine, peptide bond etc.) bonds can be engaged to attach bioactive agents [15, 16].

Gelsolin (GSN) belongs to a family of proteins that have the ability to bind and shorten actin filaments and are regulated by phosphoinositides such as phosphatidylinositol 4,5 bisphosphate (PIP_2_), sphingosine 1-phosphate (S1P) and by lysophosphatidic acid (LPA) [17]. It is composed of a chain of 730 amino acids, organized in six homologous segments G1-G6, each of which is responsible for different protein functions [18]. PBP10 peptide (GSN 160–169) is derived from the corresponding GSN PIP2-binding sequence, which, when coupled to rhodamine B, shows antibacterial activity against both Gram-positive and Gram-negative bacteria [19]. In addition, it was found that intact GSN and PBP10 bind to major constituents of the bacteria cell wall, such as lipopolysaccharides (LPS) and lipoteichoic acid (LTA) suggesting that this GSN domain might be engaged in bacteria targeting [20–22]. This possibility is reinforced by preferential gelsolin binding to structurally similar bioactive phospholipids that regulate the intracellular activity of GSN, i.e. phosphatidylinositols (PIP, PIP_2_) and. The structural similarity of LPS, LTA, LPA, and PIP2 is mostly caused by the presence of phosphorylated saccharides substituted with terminal acyl chains.

In this study we investigated the antimicrobial properties of gold and aminosilane coated magnetic nanoparticles in combination with derivatives of a PBP10 peptide including the native peptide (PBP10), a peptide with an additional amino acid, cysteine, functionalized by rhodamine B (Cys-PBP10-RhB) and a peptide functionalized by rhodamine B (PBP10-RhB). Their antibacterial activity was measured against representative bacteria (*Staphylococcus aureus* MRSA Xen 30, *Pseudomonas aeruginosa* Xen 5) and fungal cells (*Candida albicans*, *Candida glabrata* and *Candida tropicalis*). We demonstrate that co-treatment of the planktonic form of pathogens and those embedded in biofilm matrix by PBP10 derivatives in combination with magnetic nanoparticles is more effective than therapy with either of these agents alone. Moreover, we show that MNPs might modulate the antimicrobial properties of the native peptide, which in free form does not exert a bactericidal effect. These findings demonstrate magnetic nanoparticles as a promising sensitizer that can enhance the antimicrobial effects of short cationic peptides.

## Materials and methods

### Synthesis of peptide-functionalized magnetic nanoparticles

Magnetic nanoparticles were created using previously published procedures with some modification [23]. Briefly, iron-oxide cores were obtained via modification of Massart’s method, which is based on a co-precipitation procedure of iron chloride salts. Gold functionalized nanoparticles were synthesized using a modification of the K-gold procedure. Aminosilane shells were obtained by one-step polycondensation of 3-aminopropyl-methoxy silane (APTMS). In both procedures, the nanoparticles were washed three times with water and ethanol, then dried in an oven at 50 °C into a powder [24]. In the next step, nanoparticles were re-suspended in phosphate buffered saline (PBS) and each peptide derivative was added in equal concentration and incubated for nucleation and growth for 48 to 72 h [25]. The native polycationic PBP10 peptide, which represents a 10 amino acid sequence from PIP_2_ binding site of human gelsolin 160–169 (QRLFQVKGRR) and the membrane-permeant polycationic peptide PBP10 linked to rhodamine B (RhB- QRLFQVKGRR) were from Lipopharm, (Gdańsk, Poland). The peptide with an additional amino acid, cysteine, (Cys-PBP10-RhB) was a gift from Dr R. Vagners (Peptide Synthesis Laboratory, Organiskas Sintezes Instituts, Riga, Republic of Latvia).

### Characterization of peptide-functionalized magnetic nanoparticles

The chemical structure and morphology of the synthesized nanosystems were evaluated by a number of techniques. All FT-IR spectra were registered in the wavenumber range of 4000 to 500 cm^−1^ by co-adding 32 scans with a resolution of 4 cm^−1^. Differential scanning calorimetry (DSC) was recorded on a DSC Discovery (TA Instruments, USA). Nitrogen was used as a purge gas with a flow of 10 mL/min. Nanosystems (2 mg) were placed in aluminum pans and heated from 25 to 500 °C, with a heating rate of 5 °C/min. Transmission electron microscopy TEM/EDX (Tecnai G2 X-TWIN) was used to characterize the morphology of the MNPs. SEM images were obtained using a scanning electron microscope (FEI Inspect S50). Crystalline (XRD) analysis was made using an X-ray diffractometer (Bruker D8 Advanced).

### Antibacterial analysis

The antibacterial activity of agents alone (PBP10, Cys-PBP10-RhB, and PBP10-RhB) or in combination with MNPs (MNP@Au and MNP@NH_2_) were tested against *P. aeruginosa* Xen 5 and *S. aureus* Xen 30. Bacteria were grown to mid-log phase at 37 °C, then resuspended in LB and brought to 10^9^ CFU/mL (OD_600_ ~ 0.5–0.8). Next, 100 µL of bacteria suspension were added to each well at a concentration range of 5–50 µg/mL. Changes in bacterial chemiluminescence intensity were monitored within 1 h and performed using a Labsystem Varioscan Lux (Thermo Scientific). *P. aeruginosa* Xen 5 and *S. aureus* Xen 30 were incubated with different concentrations (5, 10, 20 and 50 µg/mL) of compounds to evaluate their effect on the formation of biofilm. After 48 h incubation, the medium was removed and the wells were washed in order to remove planktonic cells. Biofilm mass was stained using 0.1% (w/v) crystal violet (CV). After a 15 min incubation, the unbound dye was removed, and the plates were thoroughly rinsed. Biofilm mass was spectrophotometrically measured at a wavelength of 580 nm and the results were compared with the values obtained in the control.

### Antifungal testing

To evaluate the fungicidal activity, killing assays were performed against clinical strains of *Candida* spp. (*C. albicans*, *C. glabrata* and *C. tropicalis*) isolated from a patient diagnosed with local mycosis as described previously [26]. Briefly, *Candida* cells were grown to mid-log phase at 37 °C, re-suspended in PBS, and brought to 10^8^ CFU/mL (OD_600_ = 0.5) and then 100 µL was added to 10 mL of PBS. Fungal cells were then added to PBS containing different concentrations agents and incubated 1 h at 37 °C. Next, the plates were transferred to ice, and suspensions were diluted 10- to 1000-fold in PBS. 10 μL aliquots were spotted on Sabouraud dextrose agar plates for overnight culture at 37 °C for CFU determination. In another set of experiments, a resazurin based assay was performed. Changes in fluorescence (ex520/em590 nm) during a 5 h incubation of *Candida* spp. (OD_600_ = 0.1) in the presence of the tested peptide in free and immobilized forms (at a concentration range of 10–50 μg/mL) were measured as an additional method to assess cell proliferation and kinetics of growth (Labsystems Varioscan Lux, Thermo Scientific).

To assess the ability to prevent the formation of biofilm, *Candida* spp. cells were grown for 48 h at 37 °C in the presence of peptide derivatives. After incubation, each well was washed with PBS to remove planktonic cells. Biofilm mass was evaluated using the crystal violet (CV) staining (0.1%) method. The unbound stain then was rinsed out with water and extracted with ethanol (70%, 100 μL), then the optical density of extract (OD) was measured at 580 nm.

### Atomic force microscopy (AFM)

A clinical isolate of *C. albicans* was resuspended in distilled water (OD_600_ = 0.15) and incubated with PBP10 derivatives in free and immobilized forms at 20 μg/mL. Then, 50 µL was spotted on a mica surface that was previously functionalized by 5% (3-aminopropyl) triethoxysilane (APTES) until completely dry. Atomic force microscope (AFM) measurements were performed directly. Images were collected using a Nano Wizard 4 BioScience AFM (JPK Instruments, Germany) working in contact and Quantitative Imaging (QI) mode. MLCT (Bruker) triangular pyramid-shaped tips with a nominal spring constant equal to 0.1 N/m were employed. Initially, the tip was brought into contact with the surface of a fungal cell until a given deflection of the cantilever was reached. The scanning was then started with a constant velocity of 2 µm/s. The three signals were recorded simultaneously while scanning the sample surface: topography, vertical deflection and lateral deflection of the cantilever, with a resolution of 128 pixels per line. Topography images serve as a qualitative assessment while vertical and lateral deflection uncover surface features with better clarity. Due to the softness of fungal cell after incubation and lateral forces during contact mode scanning, a force curves-based imaging mode was used (QI mode) with the resolution of 128 pixels per line, using the same cantilever. We only show images from vertical deflection and QI mode.

### Inner membrane (IM) permeabilization assay

IM permeabilization was evaluated by measuring the release of cytoplasmic β-galactosidase activity from *Candida* spp. using ONPG (*ortho*-nitrophenyl-β-d-galactopyranoside) as the substrate [27]. Fungal cells were resuspended in a PBS (OD 600 = 0.1). Next, to 100-μL of *Candida* cells, various concentrations of agents (5, 10 and 50 µg/mL) were added and mixed with 10 μL of ONPG (30 mM) in a 96-well plate. The production of *o*-nitrophenol during 2 h was determined by monitoring the change in absorbance at 420 nm using Labsystem Varioskan Lux (Thermo Scientific) spectrophotometer.

## Results

### Synthesis and physicochemical properties of peptide functionalized magnetic nanoparticles

**Fig. 1:**
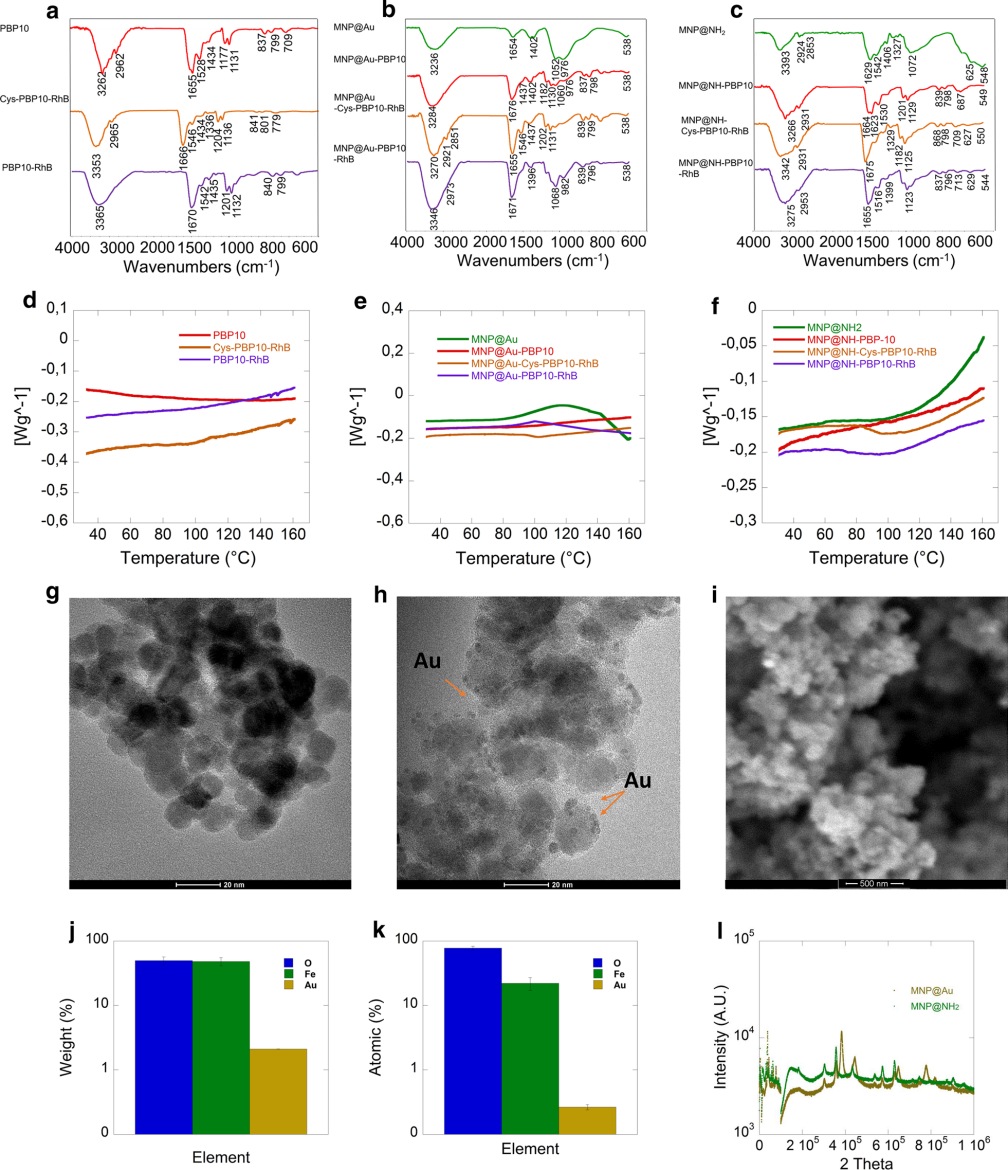
Physicochemical properties of magnetic nanoparticles functionalized by derivatives of PBP10 peptides. ATR/FT-IR spectra of PBP10 derivatives. **a** Spectra of peptides in non-immobilized form. **b**, **c** Spectra of derivatives of PBP10 peptides anchored on gold modified (**b**) and aminosilane coated-magnetic nanoparticles. **d**–**f** Thermal properties of evaluated compounds (d-thermogravimetric curves of peptides in free form and **e**, **f**—as immobilized on gold modified and aminosilane coated-magnetic nanoparticles respectively). TEM image of aminosilane coated (**g**) and gold modified (**h**) magnetic nanoparticles (Au nanoparticles on the nanoparticle surface are indicated by orange arrow). SEM images of Au coated magnetic nanoparticles (**i**). Analysis of element weight (**j**) and atomic structure (**k**) of gold modified magnetic nanoparticles. X-ray diffractogram of gold modified and aminosilane coated magnetic nanoparticles (**l**)

In this study peptide immobilization on the surface of magnetic nanoparticle was successfully performed in a one-pot process. As a result, nanoparticles with a spherical shape, relatively low polydispersity and high incorporation of peptides were prepared. PBP10 peptides were attached to the nanoparticle surface via non-covalent interactions including electrostatic interactions or dative binding. The presence of an iron oxide magnetic core in all nanosystems is indicated by a band around 538–550 cm^−1^, which can be ascribed to the Fe–O stretching mode of magnetite (Fe_3_O_4_). The broad band at ~ 970–1130 cm^−1^ corresponds to the stretching vibrations of organosilane derivatives including: Si–O, Si–O–Si and Si–O–Fe. However, after the attachment of peptides, these peaks were overlapped by signals characteristic of PBP10 derivatives. The signals above 3200 cm^−1^ are characterized by N–H stretching vibrations. The strong adsorption signals at ~ 1660 cm^−1^ represent the spectrum of peptides in free and immobilized form and can be assigned to the C=O stretching bond. The bands observed in the area of 1200–1450 cm^−1^ were assigned to OH deformation mode. The signals recorded at 840–640 cm^−1^ correspond to out of plane NH bending. The signal characteristic of S-S bonds can be formed, and signal for this group can be registered around 550 cm^−1^. In our case, in this area, strong Fe–O vibrations exist and overlap this weak peak. The thermal properties of peptides, in free and immobilized forms, were characterized by differential scanning calorimetry—DSC (Fig. 1d–f). The heating curves registered for peptides and aminosilane- and gold-coated MNPs indicate differences in the chemical nature of the coating. Curves shown in panels E and F indicate that the presence of peptide derivatives determines the course of the nanosystem heating curve. This result supports the data obtained by FT-IR spectroscopy and provides some evidence of a successful immobilization procedure. To evaluate the size and the morphology of the nanoparticles we used transmission electron microscopy. The particles were found to be spherical, and average size was estimated at 12–13 nm for aminosilane- and gold-coated MNPs respectively (Fig. 1g, h). These results were also confirmed by the XRD technique (calculated by the Williamson–Hall method) (Fig. 1l). Additionally, to detect the presence of gold on the surface of synthesized nanoparticles, SEM/EDX analysis was performed. This assay indicated that oxygen, iron, and gold content are 49%, 48% and 2% respectively of the nanostructure weight while the average atomic percentage in the samples was 77%, 22% and 0,27% respectively (Fig. 1j, k).

### Peptide-functionalized magnetic nanoparticles affect the metabolism and formation of bacterial biofilm

**Fig. 2:**
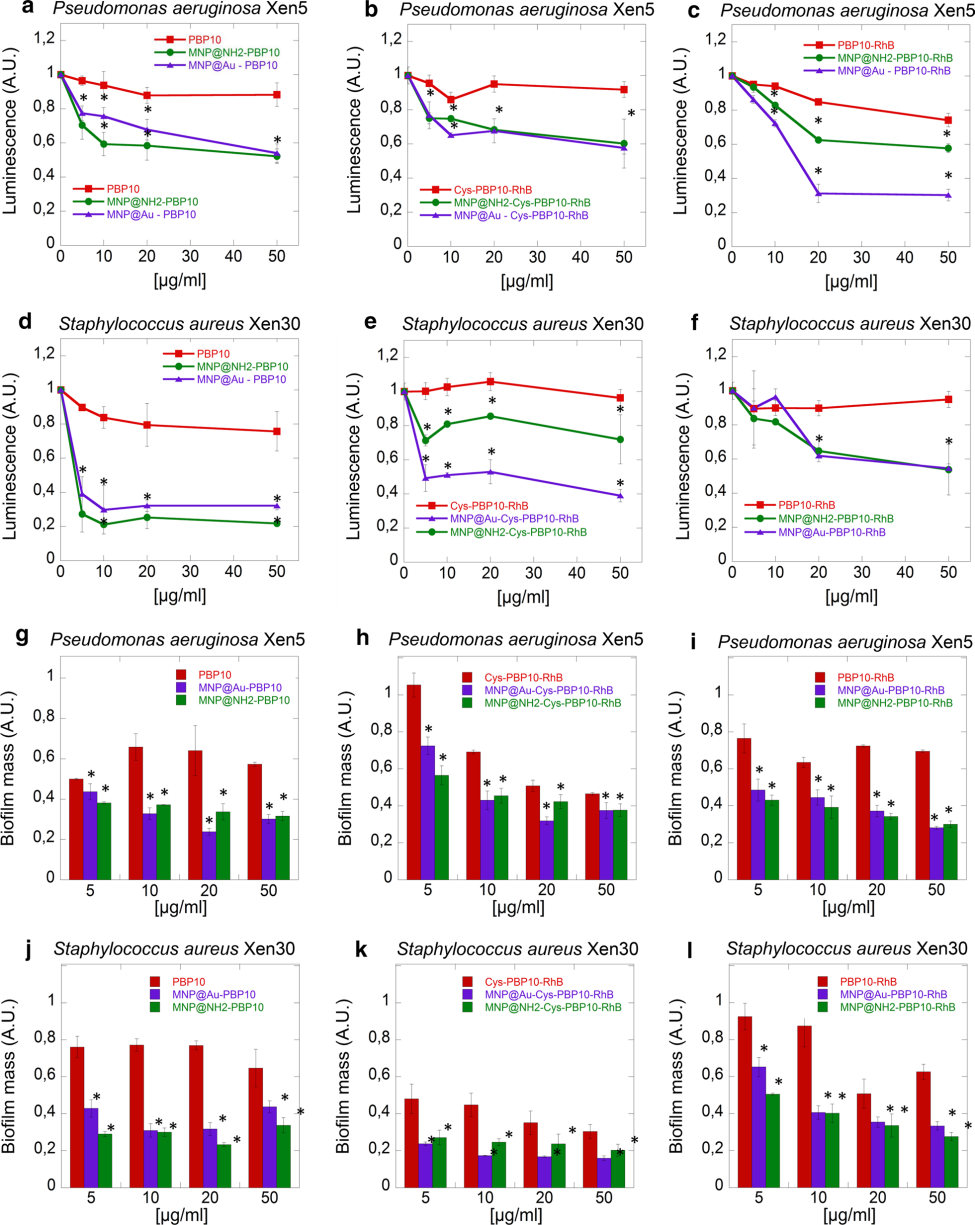
Magnetic derivatives of PBP10 peptides affect the metabolic activity of antibiotic-resistant bacteria adopting a planktonic pattern of growth and restrict biofilm formation. **a**–**f** Indicate activity of PBP10 derivatives against the planktonic form of *Pseudomonas aeruginosa* Xen 5 and S*taphylococcus aureus* (MRSA strain) respectively. Ability to prevent the formation of a biofilm of *Pseudomonas aeruginosa* Xen 5 (**g**–**i**) and MRSA (**j**–**l**). Statistical significance for the samples treated by peptides in immobilized form compared to the peptide in free form is marked by (*)

In the initial stage of the study, we assessed the ability of gelsolin-derived PBP10 peptides in free and immobilized forms to restrict bacterial metabolic activity in a short time incubation. We used bioluminescent pathogenic strains *P. aeruginosa* Xen5 and *S. aureus* Xen30, which possess a stable copy of the *luminescent* lux operon on the bacterial chromosome [28]. As shown in Fig. 2a–f, a 10–20% decrease of chemiluminescence was observed after treatment of both pathogens with PBP10 derivatives applied in free form. Importantly, in the case of a representative Gram-negative bacteria, significant metabolic inhibition, up to 40% and 70%, was observed after the addition of peptide-functionalized aminosilane and gold-coated magnetic nanoparticles, respectively. In the case of *S. aureus*, the addition of nanosystem-based, aminosilane-coated MNPs caused 30%, 50% and 80% restriction of metabolic activity for Cys-PBP10-RhB, PBP10-RhB, and PBP10 derivatives respectively. In turn, a significant decrease of chemiluminescence, up to 40%, 60% and 70% was noted after treatment by PBP10-RhB, Cys-PBP10-RhB and PBP10 derivatives anchored on gold decorated MNPs. We observed a twofold increase in activity of Cys-PBP10-RhB derivatives when attached to gold-coated MNPs compared to aminosilane coated ones, which provides evidence that the type of interaction between active agents and the shell plays an important role in developing an effective method of treatment. Together, these data suggest that the engagement of gold decorated nanoparticles to deliver peptide in the presence of a thiol group are needed to obtain better antimicrobial activity.

Next, we explored the potential of magnetic nanoparticles to increase the ability of PBP10 peptide derivatives to prevent biofilm formation. Panels g–l of Fig. 2 illustrate that PBP10 derivatives alone are able to prevent biofilm formation in both strains. However, to obtain inhibition of biofilm formation in the 30–60% range, application of a high dose of peptides (50 µg/mL) is required. Addition of nanoparticles allows us to accomplish this with smaller doses. For example, when combined with gold decorated MNPs, treatment with 10 µg/mL peptide derivatives decreases biofilm formation by 60% for all PBP10 derivatives for *P. aeruginosa*, 60% for PBP10 and PBP10-RhB, and 90% for Cys-PBP-RhB in the case of biofilm formed by *S. aureus*.

### Magnetic nanoparticles modulate fungal killing properties of PBP10 derivatives

**Fig. 3:**
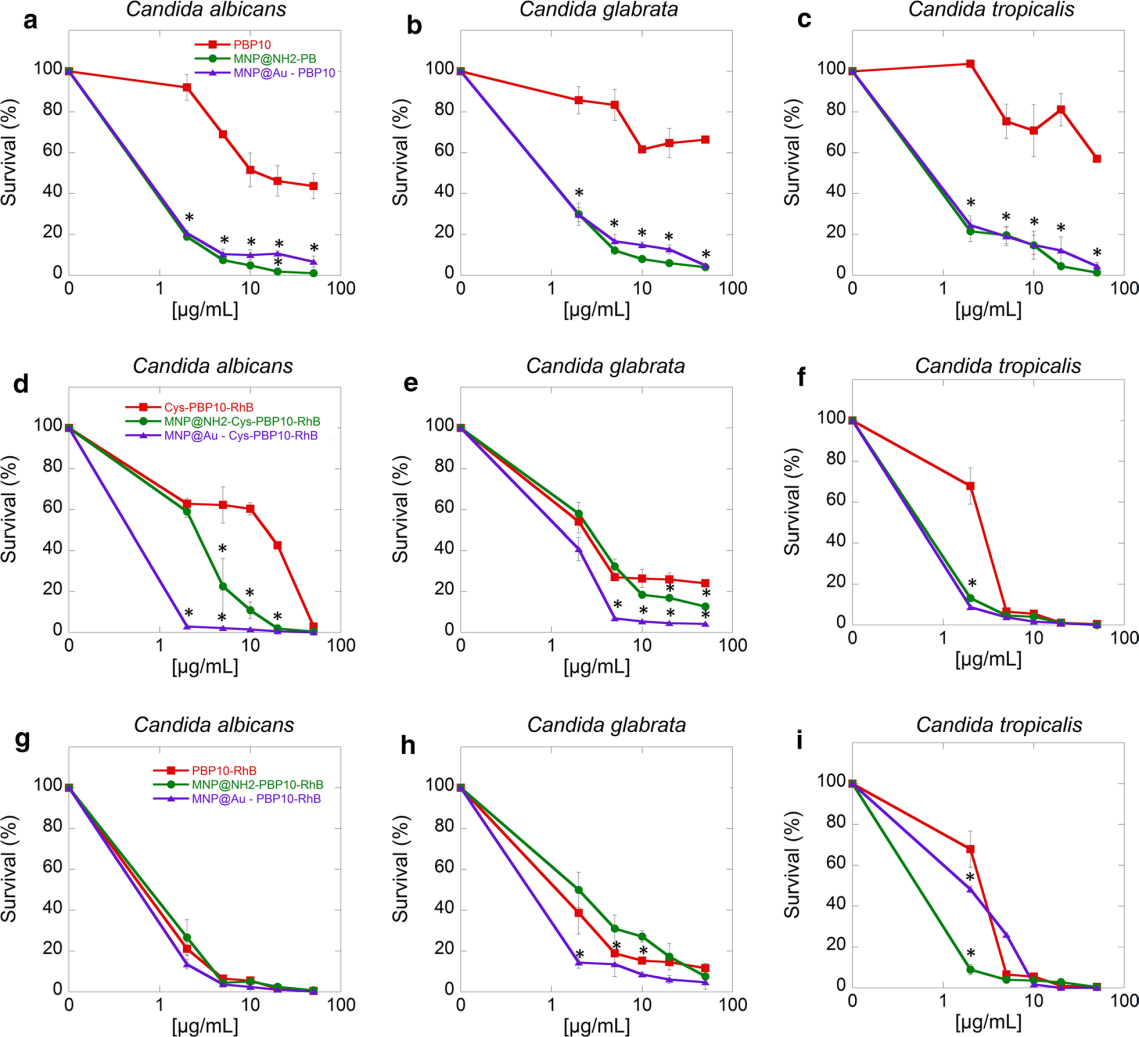
Candidacidal activity of PBP10 peptide derivatives in free and immobilized form. Killing activity of tested agents against clinical isolates of *C. albicans* (**a**, **d**, **g**), *C. glabrata* (**b**, **e**, **h**) and *C. tropicalis* (panels **c**, **f**, **i**). Statistical significance for the samples treated by peptides in immobilized form compared to the peptide in free form is marked by (*)

**Fig. 4:**
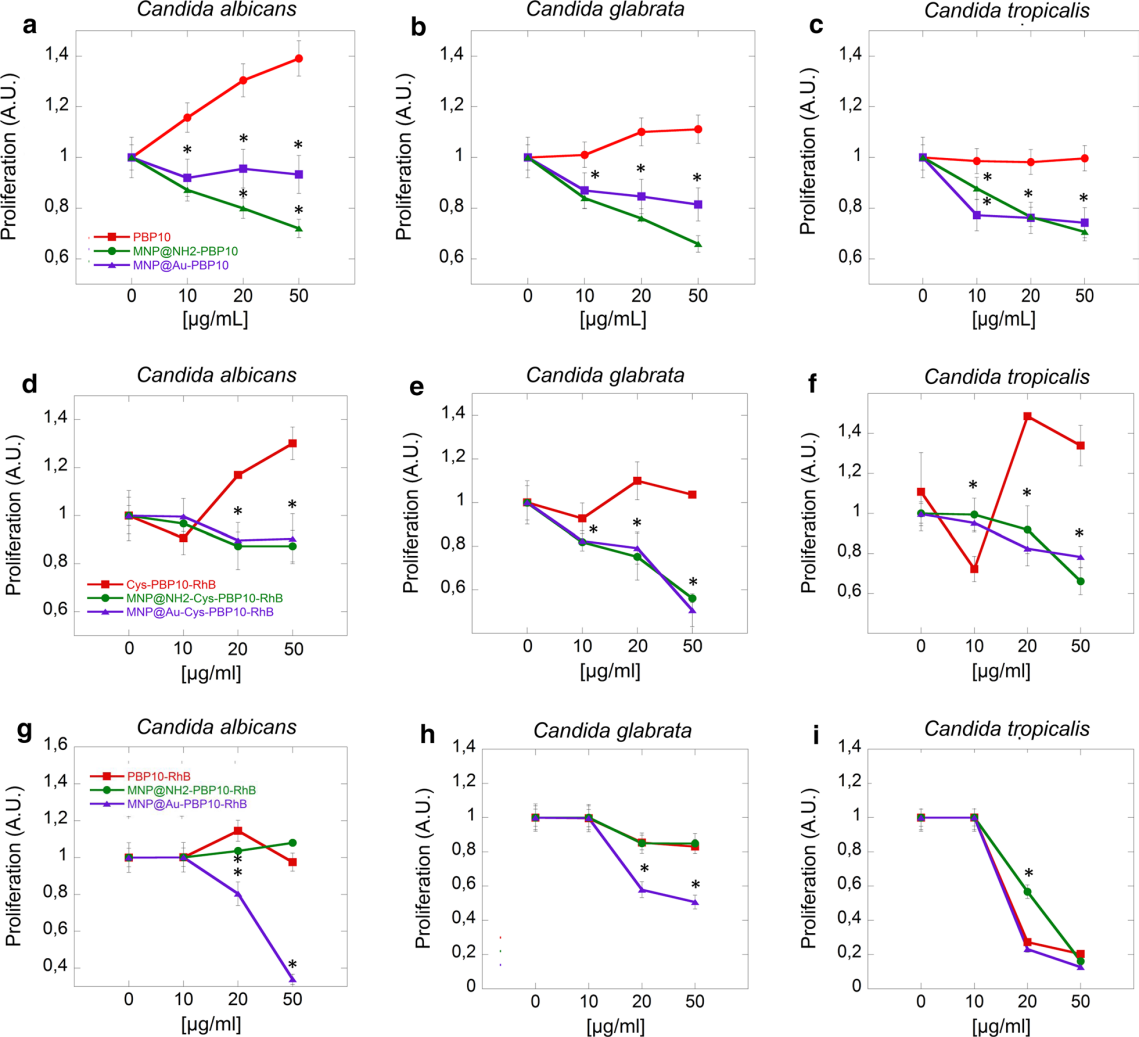
Magnetic derivatives of PBP10 peptides restrict the proliferation of *Candida cells*. The impact of PBP10 derivatives in the free and immobilized form on *C. albicans* (**a**, **d**, **g**), *C. glabrata* (**b**, **e**, **h**) and *C. tropicalis* (**c**, **f**, **i**) growth. Statistical significance for the samples treated by peptides in immobilized form compared to the peptide in free form is marked by (*)

The antifungal efficacy of PBP10 in free form and immobilized on magnetic nanoparticles against *Candida* cells was tested using a killing assay. In the case of native PBP10 peptides, magnetic derivatives were more potent and resulted in a ~ 75% decrease of cell viability at 5 µg/mL against all tested *Candida* spp. (Fig. 3a–c). Notably, application of a higher dose (50 μg/mL) of native PBP10 was insufficient to totally inhibit fungal growth, with a restriction at approximately 50%, while in the case of magnetic derivatives 100% reduction of viable cells was observed. Figure 3d–f show the killing activity of cysteine-functionalized and RhB-labelled PBP10 in free and immobilized form. The strongest restriction at more than 95% with the smallest applied dose (5 µg/mL) was observed for gold-coated peptide derivatives against all tested *Candida* spp. strains. Inhibition at a similar level, for aminosilane coated derivatives, was observed at a four times higher concentration (20 µg/mL) against *C. albicans* and *C. tropicalis* compared to gold coated ones. Fungicidal activity of Cys-PBP10-RhB applied in free form was as follows: total restriction was observed at the highest used concentration for *C. albicans* and *C. tropicalis* strains, while in the case of *C. glabrata*, 80% reduction of viability was noted. This observation provides some evidence that the type of interaction between nanoparticle surface and bioactive agents might determine the biological efficiency of the nanosystem. Killing activity of RhB-labelled PBP10 peptides and their magnetic derivatives indicated that in the case of *C. albicans* and *C. glabrata*, similar activity for both tested forms was noted (Fig. 3g, h). Treatment of *C. tropicalis* demonstrated that at the lowest applied concentration (2 µg/mL), 1.5 and 7-times better killing activity was observed for gold coated and aminosilane coated magnetic nanoparticles respectively, compared to the free peptide. However, at a concentration range 5–50 µg/mL an insignificant difference in killing the ability of all tested combinations was noted (Fig. 3i). As a confirmation of provided data, proliferation capability of treated *Candida* cells was evaluated using resazurin-based fluorescence assay. As shown in Fig. 4, immobilization of tested peptides on the surface of gold-coated and aminosilane-modified nanoparticles significantly improves their fungicidal properties. Notably, an activity of tested agents was considerably variable among tested strains; native PBP10 was effective against *C. glabrata* and *C. tropicalis* strains, but it was not able to limit the proliferation of *C. albicans* cells (Fig. 4a–c). Moreover, the rhodamine B-labeled PBP10 peptides were characterized by greater anti-proliferative features than unlabeled PBP10 (Fig. 4d–i). Importantly, for all tested peptides, magnetic nanoparticles improved killing capabilities by approx. 30% for PBP10 (Fig. 4a–c) and Cys-PBP10-RhB (Fig. 4d–f). Most notably, immobilization of PBP10-RhB on the surface of MNP@Au strongly limited proliferation of fungal cells, since 30–80% inhibition in proliferation ratio for this combination was observed (Fig. 4g–i).

### Magnetic nanoparticles increase anti-biofilm activity of tested peptides

**Fig. 5:**
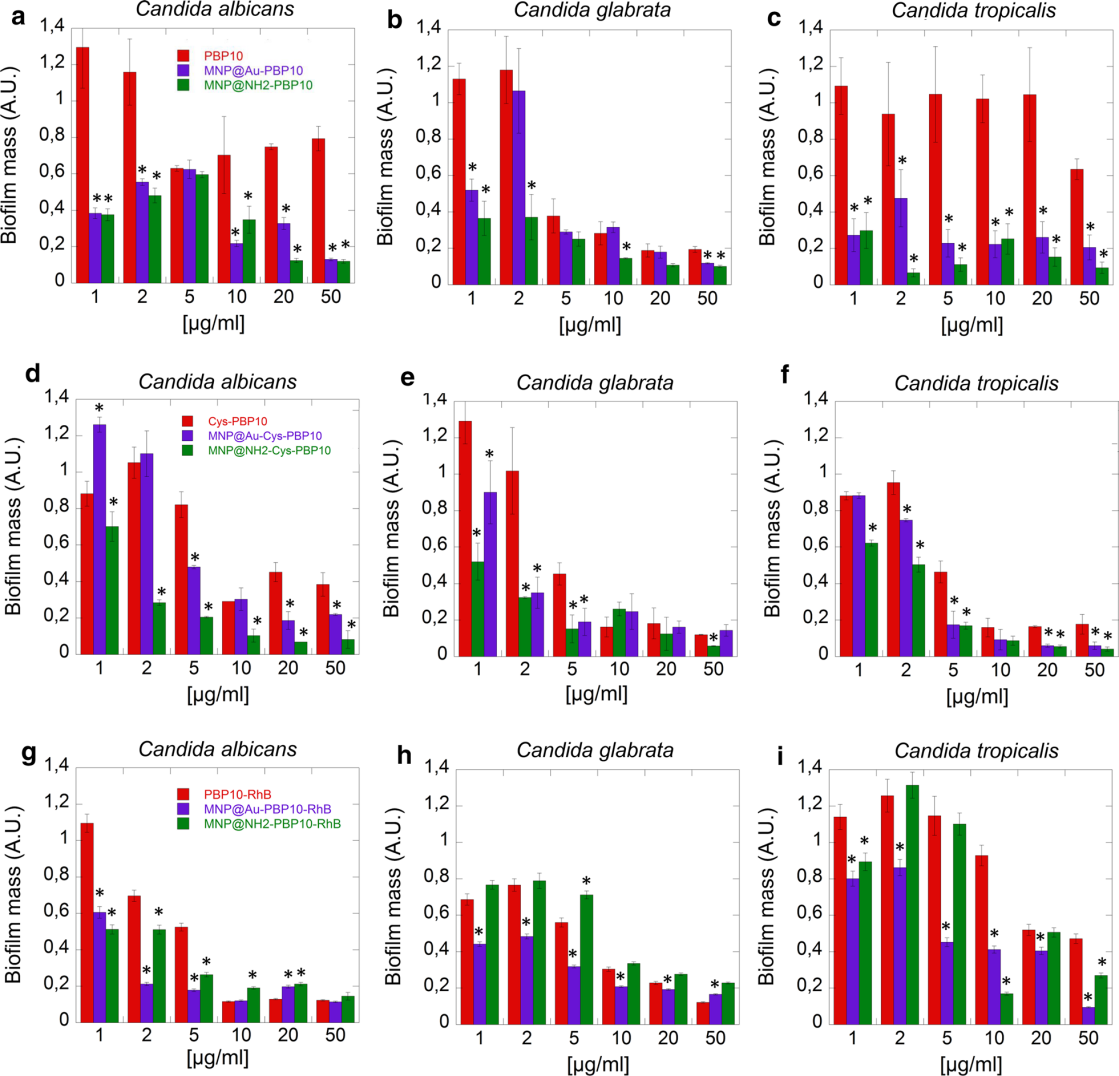
Anti-biofilm activity of PBP10 coated magnetic nanoparticles against *Candida* strains. The ability of PBP10 derivatives in the free and anchored form to prevent biofilm formation of *C. albicans* (**a**, **d**, **g**), *C. glabrata* (**b**, **e**, **h**) and *C. tropicalis* (**c**, **f**, **i**). Statistical significance for the samples treated by peptides in immobilized form compared to the peptide in free form is marked by (*)

To investigate the anti-biofilm activity of PBP10 derivatives in free and immobilized form, fungal biofilms were formed for 48 h in the presence of tested agents. Biofilm mass was then spectrophotometrically detected using the CV-staining method. In correlation to viability (Fig. 3) and proliferation measurements (Fig. 4), RhB-conjugated peptides were more effective in prevention of biofilm formation than PBP10 (Fig. 5a–c). In general, peptides in free form exert lower antibiofilm properties than their magnetic derivatives. However, in some cases, a similar effect was observed at a concentration above 20 µg/mL [particularly for Cys-PBP10-RhB (Fig. 3d–f) and PBP10-RhB (Fig. 3g–i)]. At 1–10 µg/mL a two-fold increase in restriction was noted. In the case of *C. tropicalis*, ~ 80% inhibition was observed after the addition of magnetic derivatives compared to unattached derivatives.

### Peptide-functionalized magnetic nanoparticles induce disruption of membrane integrity and leakage of cytoplasmic components

**Fig. 6:**
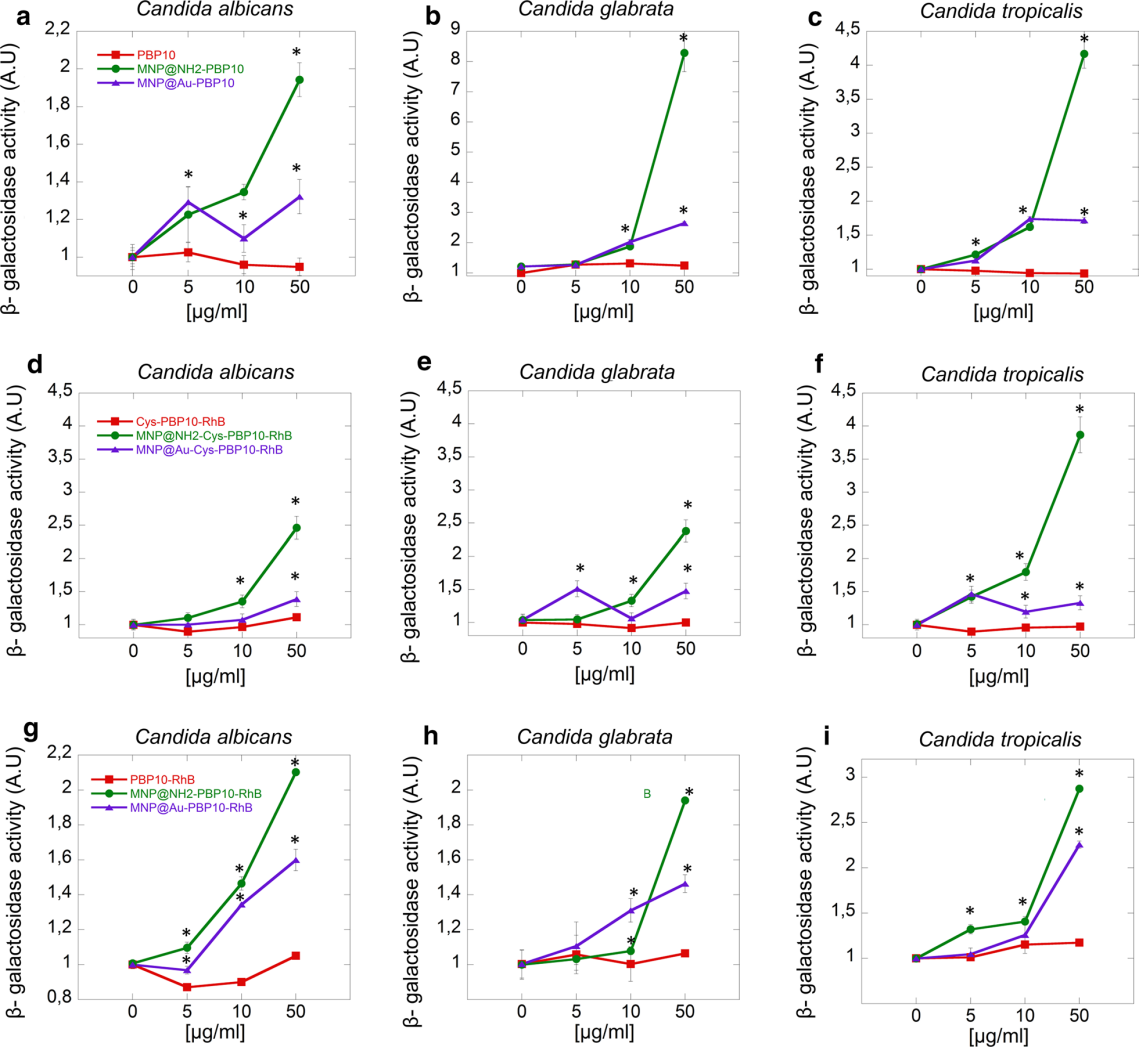
Magnetic nanoparticles disrupt fungal membrane and increase insertion of PBP10 derivatives. Increasing β-galactosidase activity after treatment of *C. albicans* (**a**, **d**, **g**), *C. glabrata* (**b**, **e**, **h**) and *C. tropicalis* (**c**, **f**, **i**) by magnetic derivatives of PBP10 peptides. Statistical significance for the samples treated by peptides in immobilized form compared to the peptide in free form is marked by (*)

In order to explore the ability of PBP10 derivatives in free and immobilized forms to cause disintegration of the fungal inner membrane, an ONPG assay was employed. Due to the fact that ONPG interacts with the cytoplasmic enzyme β-galactosidase after membrane destabilization, the formation of *o*-nitrophenol can be spectrophotometrically measured at 420 nm. Magnetic derivatives of PBP10 peptides induce major permeability changes in the inner fungal membrane, which is shown by a significant increase of β-galactosidase activity compared to peptides in free form (Fig. 6). For aminosilane derivatives, a two to eightfold increase of enzyme activity was detected. In turn, in the case of gold-coated nanoparticles, a twofold higher activity of β-galactosidase was noted. In both cases, the release of β-galactosidase was observed in a dose dependent manner. However, after treatment by free peptides, changes in membrane permeability were seen only for PBP10 derivatives functionalized by rhodamine B. Addition of native PBP10 does not exert any effect on the fungal membrane (Fig. 6a–c).

**Fig. 7:**
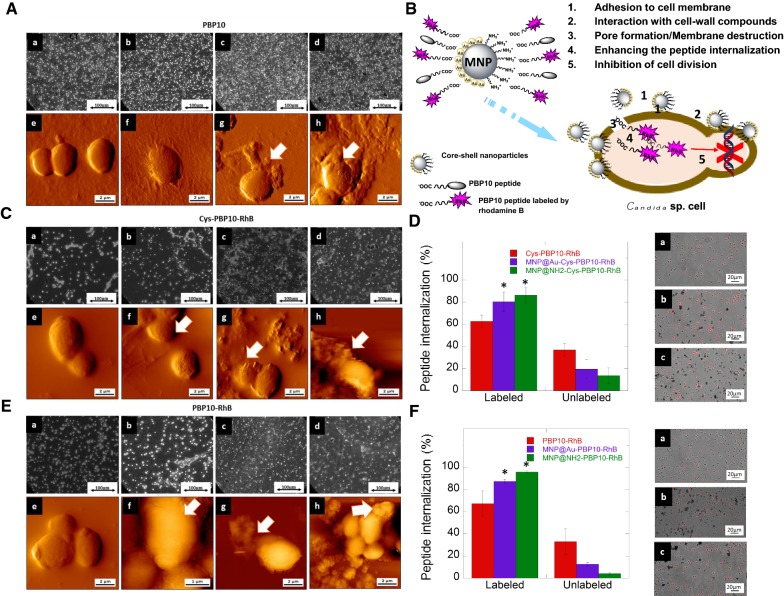
Magnetic nanoparticles enhance the uptake of PBP10 peptides by *Candida* cells and increase candidacidal properties. Optical images and atomic force microscopy topography of *Candida albicans* cells treated by tested peptides as free and immobilized molecules. *C. albicans* cells treated by unlabeled PBP10, Cys-PBP10-RhB and PBP10-RhB derivatives (**A**, **C**, **E**, respectively): a, e—control; b, f-treated by peptide alone; c, d and g, h—treated by agent immobilized on gold or aminosilane coated MNPs, respectively (e–h vertical deflection mode). **B** indicates hypothesized mode of action proposed for tested PBP10 derivatives immobilized on MNPs. **D** (Cys-PBP10-RhB) and **F** (PBP10-RhB) show internalization of peptide into *Candida albicans* cells treated by PBP10 peptides in free and immobilized form; small panels show merged photography of *C. albicans* cells: a—treated by PBP10 peptide; b, c—treated by PBP10 peptide immobilized on gold modified and aminosilane coated MNPs. Statistical significance for the samples treated by peptides in immobilized form compared to the peptide in free form is marked by (*)

Further, we investigated the effect of peptide derivatives on fungal cell morphology. To visualize their effects on the clinical isolates of the vegetative form of *C. albicans* we used atomic force microscopy. After treatment by free peptides, we observed a lack of marked effect (native PBP10) or low disruption in membrane morphology (microcracks, surface wrinkling). Compared to the free form, a significantly different effect was observed after the addition of magnetic derivatives of PBP10 peptides, characterized by a more corrugated surface on the fungal cells. Moreover, nanoparticles attach to the fungal cell surface and cover them. We suggest that this phenomenon is responsible for the formation of a crack or tiny pore on the cell surface and leads to leakage of cytoplasmic content. This result could be seen as evidence that magnetic nanoparticles sensitize the fungal cells when exposed to the peptide. As indicated by results from confocal microscopy, a higher percentage of cells take up rhodamine B labeled peptide in the presence of magnetic nanoparticles compared to those exposed to the peptide in free form (Fig. 7d, f). Together, these results indicate that magnetic nanoparticles significantly enhance the effect of PBP10 peptides through a membrane-based mode of action. They attach to the cell surface, interact with cell wall components, disrupt the membrane, increase uptake and sensitize the fungal cells to peptide treatment that results in inhibition of cell division.

## Discussion

In our study, we revealed that magnetic nanoparticles significantly elevated the sensitivity of microbial cells to the effects of PBP10 derivatives. We observed increased activity of GSN derived peptides in a killing assay against planktonic bacterial and fungal cells and a better ability to interrupt biofilm formation when anchored on the surface of nanoparticles. MNPs improved the fungicidal effect of peptides through disruption of the fungal membrane and facilitated peptide transport into cells. Moreover, the designed nanosystems prevent cell division. This finding provides evidence that nanoparticles might be a promising sensitizer of pathogenic cells, and, nanostructures have potential as enhancers of fungal infection treatment. The formulation presented in this report might be useful for creating a general pattern to improve peptide efficacy via immobilization procedure.

Currently, to treat infections, antibiotics are still the main therapeutic option [29], but misuse of antibiotics, including their massive application for prophylactic purposes without proper medical indication, causes strain selection and development of multidrug resistance patterns [30–32]. Unfortunately, antibiotic resistance is a dynamic, social and global problem, both in the case of bacterial and fungal pathogens [33]. Moreover, the number and diversity of chemical structures of currently available antibacterial drugs are much higher than the number of active substances in relation to pathogenic fungi. The appearance of MDR strains, with resistance to many groups of antimicrobial agents, creates the need to design new therapeutic strategies for successful treatment of infectious diseases [34, 35].

Antimicrobial peptides (AMPs) are a class of antimicrobial agents with a broad spectrum of protective, antimicrobial features [36]. In effect, they are a viable alternative to currently used antibiotics [37, 38]. However, they are sensitive to protease degradation and are inactivated in the presence of various polyelectrolyte factors that occur at sites of infection including F-actin, DNA and bacteriophage [26, 39, 40]. Moreover, they exert high toxicity on human host cells and cause hemolytic activity [41]. Additionally, AMPs isolated from natural sources are usually in insufficient quantity. Therefore, to obtain an adequate amount, chemical synthesis is often implemented, which is expensive [42]. These AMP disadvantages might restrict their clinical use. Due to this fact, significant efforts and different strategies have been engaged in the past decades to protect AMPs from degradation, inactivation and to improve their biocompatibility including by substitution with unnatural amino acids, d-amino acids, W or end-caps, varying chain length, cyclization or polymerization [43–45]. Another approach to increasing clinical use is through increasing stability and by decreasing their toxicity through the use of drug carriers [46]. Our previous studies have shown that both physical and covalent immobilization on the nanoparticle surface markedly enhances the activity of AMPs and their chemical analogs [23, 47]. Moreover, the embedding process prolonged in vitro peptide activity [48, 49]. Application of nanoparticles as drug carriers, during systemic injection, shows that nanosystems functionalized by homing molecules increase particle elimination with protection against non-specific organ accumulation in healthy mice as well as offer an improvement in cancer therapy due to elongated retention of MNPs in the tumor site [50]. This supports the assertion that compared to a traditional form of therapy, use of nanoparticles as a drug delivery system improves the pharmacokinetic profile of the active substance, leads to enhancement of the anticancer/antimicrobial effect, and reduces side effects and systemic toxicity [51].

Herein, we propose the application of nanoparticles as a drug delivery system for PBP10 peptides. Those fragments possess unique properties including positive charge and hydrophobicity, which are enhanced by to rhodamine B conjugation. RhB-functionalized derivatives enter passively into cells and disrupt cellular pathways that depend on phosphoinositide signaling. However, un-functionalized PBP10 derivatives do not exert the above effects, but if anchored to nanoparticles, desired targets can be achieved. The pathogen-killing process is rapid and involves the destruction of the plasma membrane, disruption of metabolic pathways and inhibition of proliferation. Furthermore, our studies show that synthesized nanosystems are more efficient than free peptide. PBP10 derivatives’ mode of action does not include pore formation during interaction with bacterial membranes, despite their similarity to antimicrobial peptides through the following features: short sequence, net positive charge, helical structure and amphipathic nature [19, 52]. PBP10 targets the bacteria cell wall via interaction with LPS and LTA due to the chemical similarity of these compounds to PIP_2_. In the case of nanoparticles, their target points involved in microbial killing include internalization in the pathogen cell wall and membrane, generation of reactive oxygen species, and creation of small pores in the bacteria cell wall [53–56]. This process enhances the uptake of active agents, oxidative damage of macromolecules and oozing of cytoplasmic content [57–59]. Therefore besides the fact that they can be used for the delivery of conventional or novel active agents, they possess intrinsic antimicrobial activity. Published data show that they are able to avoid drug resistance mechanisms in bacterial and fungal cells via different strategies including: (i) inhibition of the activity of efflux pumps; (ii) restriction of biofilms formation due to penetration and/or induction of hyperthermia processes; (iii) interference of quorum sensing; (iv) inactivation of enzyme and (v) possibly plasmid curing [60–63]. Moreover, our recent study demonstrated that PBP10-containing nanosystems exert strong anti-inflammatory and immunomodulatory activities with the ability to limit the LPS-induced cellular effects, which additionally strengthen the potential of PBP10 magnetic derivatives as protective antimicrobial agents [64].

Additionally, the presence of peptide on the surface of magnetic nanoparticles is crucial in nanosystem stabilization. Based on the findings here, we suggest that PBP10 derivatives can act as a capping agent. This kind of compound plays a key role in the inhibition of particle aggregation; in effect, they control the particle growth and structural stability [65]. The above features are required to preserve nanosystem properties in the presence of body fluids, potentiate their antimicrobial activity and enhance active molecules delivery [66].

In conclusion, the data presented in this study clearly indicate that magnetic nanoparticles enhance the antimicrobial properties of GSN derived peptides. We showed that type of interaction (electrostatic/chemisorption) between the nanoparticle surface and the bioactive agents might determine the biological efficiency of the nanosystem. Additionally, we suggest that synthesized nanoparticles could be used as a sensitizer for fungal cells to enhance the therapeutic effect of peptides. This nanoformulation might create new approaches and provide a general platform for developing new treatment options against microbial infection.

## Data Availability

Materials described in the manuscript, including all relevant raw data, will be freely available to any scientist wishing to use them for non-commercial purposes upon request via e-mail with the corresponding author.
